# Effect of sterilization on the canine vaginal microbiota: a pilot study

**DOI:** 10.1186/s12917-020-02670-3

**Published:** 2020-11-23

**Authors:** Ada Rota, Michela Corrò, Ilaria Patuzzi, Chiara Milani, Stefania Masia, Eleonora Mastrorilli, Sara Petrin, Alessandra Longo, Angela Del Carro, Carmen Losasso

**Affiliations:** 1 Department of Veterinary Sciences, University of Turin, Largo Paolo Braccini 2-5, 10095 Grugliasco, Italy; 2 Istituto Zooprofilattico Sperimentale delle Venezie, Viale dell’Università 10, 35020 Legnaro, PD Italy; 3 Department of Animal Medicine, Production and Health, Viale dell’Università 16, 35020 Legnaro, PD Italy

**Keywords:** Dog, Sterilization, Vaginal microbial community, Metataxonomic analysis, Bacteria culture

## Abstract

**Background:**

Surgical sterilization is the most effective method of contraception for dogs. It also prevents pyometra and reduces the risk of mammary tumour development. However, this procedure also has negative effects, such as urinary incontinence. Steroid hormone deprivation following gonadectomy could also affect canine vaginal mucosa conditions and the microbial community colonizing the vaginal tract. This hypothesis was tested by comparing the vaginal cytology and microbial community of two groups of bitches, including 11 in anoestrus and 10 sterilized bitches (post-pubertal sterilization in the last 4 years). Bacteria were identified through metataxonomic analysis, amplifying the V3-V4 regions of *16S rRNA* gene, and culturing methods.

**Results:**

Vaginal mucosa cytology was suggestive of dystrophic conditions in sterilized bitches, whereas a typical anoestrus pattern with parabasal and intermediate cells was appreciable in anoestrous animals. Metataxonomic analysis revealed large inter-individual variability. *Salmonella*, *Mycoplasma* and *Staphylococcus* were present in moderate quantities in almost all the samples in both groups. Mollicutes (class level) and Tenericutes (phylum level) were commonly present in moderate quantities in anoestrus samples, whereas these microbes were present at high levels in a single sample from the sterilized group.

Based on culturing, a higher number of different species were isolated from the anoestrous bitches, and *Mycoplasma canis* was exclusively identified in an anoestrous bitch. *Staphylococcus* spp*.* was the most frequently isolated genus in both groups, followed by *Streptococcus* spp., and, among gram-negative bacteria, *Escherichia* spp*.* and *Haemophilus* spp*.* A comparison of the numbers of the most frequently isolated genera of bacteria from vaginal cultures of bitches revealed that *Pasteurella* and *Proteus* were the most frequently identified in sterilized animals based on metataxonomic analysis (*p*-value = 0.0497 and 0.0382, respectively), whereas *Streptococcus* was significantly and most frequently isolated from anoestrous bitches using culture methods (*p* value = 0.0436).

**Conclusions:**

In this preliminary investigation, no global patterns of the vaginal bacteria community were noted that characterized the condition of the bitches; however, cytology suggested local modifications. Sterilization after puberty caused minimal alterations in the vaginal microbial community of bitches within 4 years after surgery.

**Supplementary Information:**

The online version contains supplementary material available at 10.1186/s12917-020-02670-3.

## Background

Surgical sterilization is currently the most effective method of contraception for dogs and has the benefits of reducing the risk for developing mammary tumours and preventing disorders mediated by ovarian hormones, such as pyometra, vaginal hyperplasia or pseudopregnancy [1]. However, in addition to its health-promoting effects, removal of the ovaries represents an alteration of physiological conditions, and the possible negative effects of sterilization have become the object of several investigations in recent years. The association between sterilization and urinary incontinence has long been known [2], whereas increased susceptibility to some orthopaedic pathologies, such as hip dysplasia or cranial cruciate ligament tear, and the development of tumours, such as lymphosarcoma or haemangiosarcoma, has recently been described in some dog breeds [3]. Longer ovary exposure is associated with a longevity advantage in a female Rottweiler dog population [4]. Thus, elective sterilization should be chosen on the basis of the balance between positive and possible negative effects.

Oestrogen loss in menopausal women causes vulvovaginal atrophy, which is commonly complicated by inflammation in atrophic vaginitis, and modifications of the lower urinary tract, which results in urinary incontinence and urinary tract infections [5]. In postmenopausal women, the immunity of vulvovaginal tissues is altered, and vaginal bacterial communities are modified [6].

In bitches, the trophic effect of oestrogen on the vaginal vasculature and muscular layer as well as on the vaginal and urethral mucosa ceases following sterilization. Anatomical differences were found in the lower urogenital tract between sterilized and intact bitches, and measurements of different tracts tended to be greater in intact animals [7]. Alterations of local conditions are likely to affect resident flora composition, but the bacteria populations found in the genital tract of bitches following sterilization have not been investigated. Detection of bacteria in the canine vaginal tract is a normal condition [8, 9]. The vaginal bacteria population of healthy dogs consists of aerobic and anaerobic microorganisms, including opportunistic pathogens, such as *Escherichia coli*, *Pseudomonas* spp., *Klebsiella* spp. [10, 11], *Mycoplasma* and *Ureaplasma* spp. [12]. Reproductive disorders and reproductive tract infections are characterized by the proliferation of microorganisms that normally belong to local bacterial communities, so the interpretation of culture tests must rely on quantitative results [13, 14].

Compared to culture-based studies, metataxonomic investigations brought to light a much more complex microbiota in the bitch vagina and detected the presence of a large number of uncommon or previously uncultured microorganisms [15]. These preliminary studies are worthy of support by further investigations.

The objective of this pilot study was to assess the effect of hormonal deprivation on vaginal mucosa and local bacteria populations in bitches. Specifically, vaginal cytology, culture results and metataxonomic analysis data were compared between healthy sterilized and intact anoestrous bitches.

## Results

### Vaginal cytology

Vaginal smears of sterilized bitches were scarcely cellular possibly as a result of tissue dystrophy following gonadal hormone deprivation. The fragility of the mucosal layer was also suggested by the presence of erythrocytes in half of the samples, which were never observed in anoestrous bitches. Vaginal smears of anoestrous bitches exhibited a normal cellular pattern with a majority of parabasal and intermediate cells and some neutrophils. Neither oestrogenic stimulation nor pathological traits were observed in any sample.

### Microbiota profiling and diversity analysis

In total, 1,900,411 sequences were obtained for the entire experiment with a mean per sample equal to 45,247.88 (SD: 65,045.2). After sequencing, raw reads were pre-processed to remove low-quality bases as well as short and chimeric sequences. After the quality trimming step, a total of 204,038 sequences were retained for further analysis with a mean of 4858 (SD: 5223) sequences per sample.

**Fig. 1:**
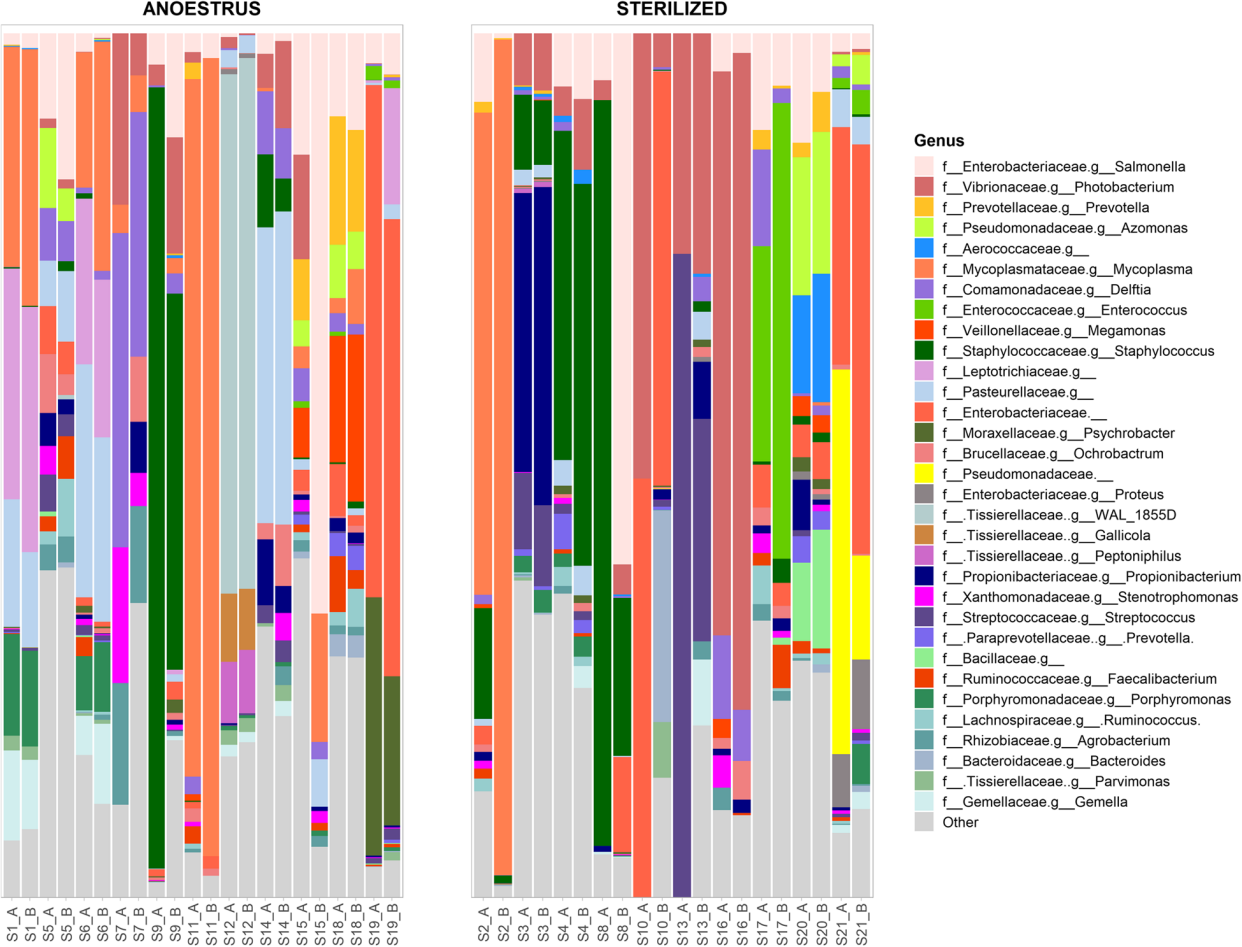
Microbial community composition (genus level) of samples from anoestrous (left) and sterilized (right) bitches. Each sample (S*) is present as two biological replicates (“_A” or “_B”)

Figure 1 presents the internal distribution of the community in terms of the most relevant genera. The first important finding is the reproducibility of sequencing results. Indeed, in the vast majority of samples, the two biological replicates led to comparable results (Fig. 1), indicating that only reasonable biological variability was observed.

Considering mean proportional abundance values, the five most present genera in anoestrus bitches were *Mycoplasma* (13.90%), an unidentified genus belonging to the *Pasteurellaceae* family (from here on called PF; 7.84%), *Salmonella* (7.60%), *Staphylococcus* (6.80%) and an unidentified genus belonging to the *Enterobacteriaceae* family (from here on called EF; 6.27%). The same parameter was applied to samples from sterilized bitches, and similar although marginally different results were obtained. Indeed, the five most relevant genera for this group were *Photobacterium* (14.03%), *Staphylococcus* (11.19%), EF (10.01%), *Mycoplasma* (7.64%) and *Salmonella* (6.82%). However, if we considered median values instead of the mean, we observed a slightly different situation. Indeed, the five most relevant genera were *Salmonella* (2.63%), *Mycoplasma* (2.17%), PF (1.86%), *Delftia* (1.60%) and EF (0.77%) for anoestrus bitches and *Photobacterium* (3.42%), *Salmonella* (3.36%), EF (1.12%), *Staphylococcus* (1.04%) and *Propionibacterium* (0.73%) for sterilized bitches.

ANCOM tests performed at each taxonomic level revealed no statistically significant differences in taxa abundances between groups at the genus, family or order level, whereas a significant difference in Mollicutes (class level) and Tenericutes (phylum level) abundance was observed (Supplementary Table 1). Indeed, these taxa were commonly present in anoestrus samples in moderate quantities, but a large number of these taxa were noted in a single sample (S2) from the sterilized group.

**Fig. 2:**
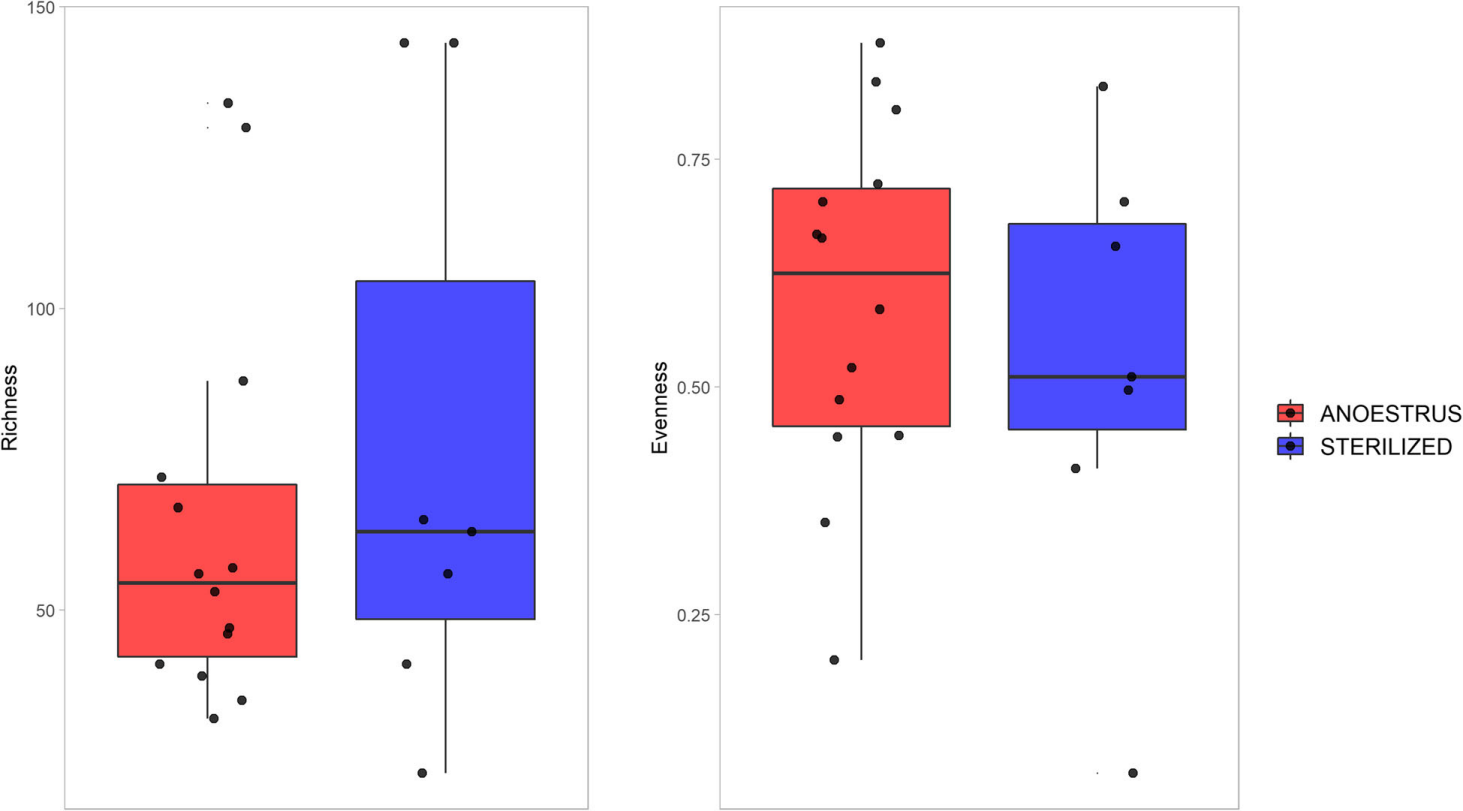
Boxplots presenting alpha diversity values calculated in anoestrus (red) and sterilized (blue) samples. On the left, a plot of richness (observed ASVs) values is reported, whereas the evenness Pielou index is presented in the plot on the right

**Fig. 3:**
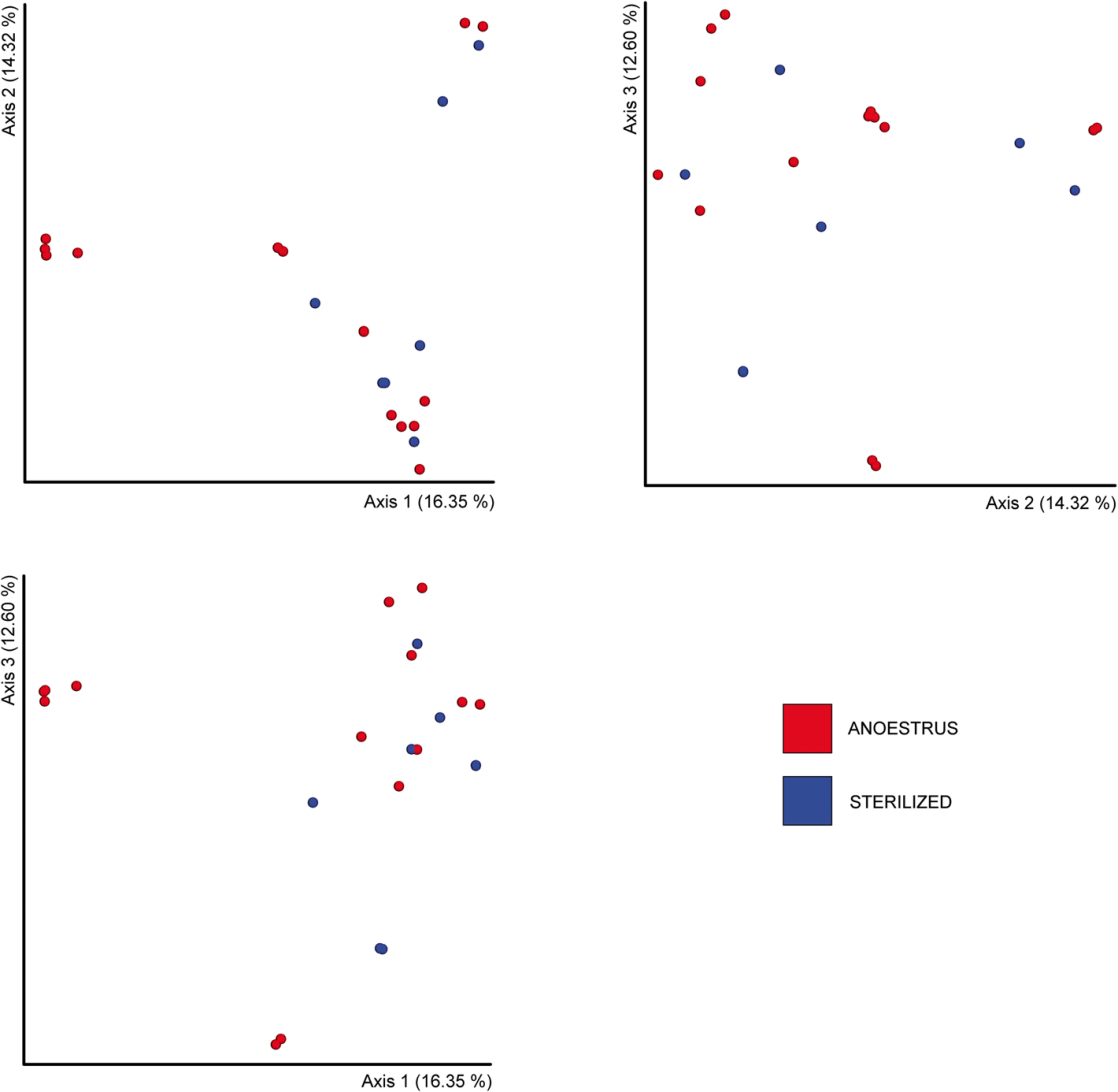
2d plots of the first three PCoA components based on Bray-Curtis distance. Anoestrus samples are shown in red, and sterilized samples are presented in blue

Diversity analysis revealed no statistically significant differences between the two groups with regard to alpha (Fig. 2; *p*-values for Kruskal-Wallis on richness and evenness: 0.4783 and 0.5019) or beta (Fig. 3; *p*-value for PERMANOVA test: 0.089) measures. Indeed, great differences were observed between single samples, but these differences were no longer observable when comparing groups (anoestrus/sterilized).

If we compare the number of anoestrous and sterilized bitches in which bacteria of genera frequently isolated from vaginal cultures were found, *Pasteurella* and *Proteus* were more frequently detected in sterilized animals (Table 1).

**Table 1:** Comparison between the number of anoestrous and sterilized bitches in which each selected genus was identified (metataxonomic analysis). The percentages are reported in parenthesis. In the third column, the *P*-values obtained from chi-square tests are reported

Genus (Metataxonomic analysis)	Anoestrus	Sterilized	***P***-value
	*N* = 11 (%)	*N* = 10 (%)	
*Bacillus*	1 (9%)	2 (20%)	0.4755
*Corynebacterium*	5 (45%)	5 (50%)	0.835
*Enterococcus*	3 (27%)	3 (30%)	0.8901
*Escherichia*^*a*^	–	–	–
*Haemophilus*	0	2 (20%)	0.1189
*Klebsiella*	0	1 (10%)	0.2825
*Lactobacillus*	6 (55%)	4 (40%)	0.5051
*Micrococcus*	1 (9%)	2 (20%)	0.4755
*Pasteurella*	0	3 (30%)	0,0497
*Pediococcus*	1 (9%)	0	0.3286
*Proteus*	1 (9%)	5 (50%)	0.0382
*Pseudomonas*	5 (45%)	5 (50%)	0.835
*Staphylococcus*	9 (82%)	10 (100%)	0.1563
*Streptococcus*	9 (82%)	8 (73%)	0.9156
*Trueperella*	1 (9%)	1 (10%)	0.9435

^a^Greengenes database does not discriminate *Escherichia* from *Shigella* genera

### Bacteria culture

Bacteriological examination revealed that the microbial species isolated from culture media generally had a very low number of colony-forming units (CFU/10 μl). Specifically, 64% of the isolates had growth of less than 5 CFU; 20% between 5 and 12 CFU; 16% greater than 30 CFU.

**Fig. 4:**
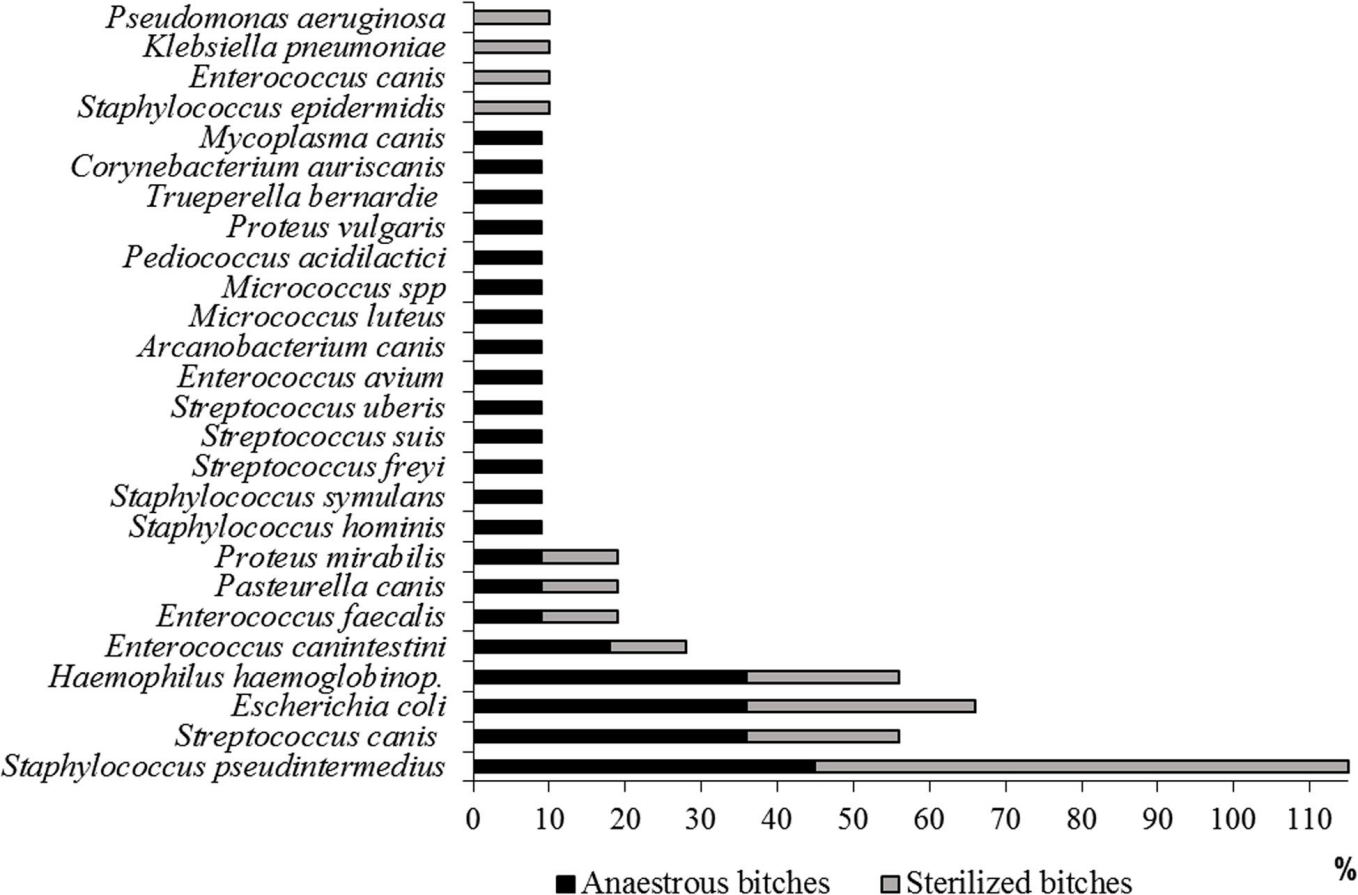
Frequency of isolation of different bacteria species in the two groups of healthy bitches

*Staphylococcus spp. (S. pseudintermedius)* was the most frequently isolated genus in both groups of animals followed by *Streptococcus spp. (S. canis).* Among Gram-negative bacteria*, Escherichia spp. (E. coli)* and *Haemophilus spp. (H. haemoglobinophilus)* were the most prevalent. As shown in Fig. 4, an increased number of different species was isolated from the eleven anoestrous bitches compared with the ten sterilized one (22 vs 12) and *Mycoplasma canis* was also identified only in the sample of an anoestrous bitch.

The isolated bacteria (*E. coli, S. canis, Arcanobacterium canis, Enterococcus* spp.) exhibited high growth in culture (greater than 50 CFU) in one anoestrous bitch and four out of ten sterilized animals.

In contrast to metataxonomic analysis results, no differences in *Pasteurella* and *Proteus* were noted, whereas the genus *Streptococcus* was significantly more frequently isolated from anoestrous bitches (Table 2).

**Table 2:** Comparison between the number of anoestrous and sterilized bitches in which each genus was found (bacteria culture). The percentages are noted in parenthesis. In the third column, the *P*-values were obtained using a chi-square test

Genus (Bacterial culture)	Anoestrus	Sterilized	***P***-value
	***N*** = 11 (%)	***N*** = 10 (%)	
*Arcanobacterium*	1 (9%)	0	0.3286
*Bacillus*	1 (9%)	0	0.3286
*Corynebacterium*	0	1 (10%)	0.2825
*Enterococcus*	4 (36%)	3 (30%)	0.7574
*Escherichia*	4 (36%)	3 (30%)	0.7574
*Haemophilus*	4 (36%)	2 (20%)	0.4071
*Klebsiella*	0	1 (10%)	0.2825
*Micrococcus*	2 (18%)	0	0.1563
*Pasteurella*	1 (9%)	1 (10%)	0.9435
*Pediococcus*	1 (9%)	0	0.3286
*Proteus*	2 (18%)	1 (10%)	0.5926
*Pseudomonas*	0	1 (10%)	0.2825
*Staphylococcus*	7 (64%)	9 (90%)	0.1566
*Streptococcus*	7 (64%)	2 (20%)	0.0436
*Trueperella*	1 (9%)	0	0.3286
*Mycoplasma*	1 (9%)	0	0.3286

## Discussion

Anoestrus is the physiological condition in which vaginal mucosa is quiescent, whereas sterilization causes the permanent loss of hormonal stimulation. The effects of the absence of oestrogen on the vaginal tract are well known in humans. Specifically, vaginal atrophy is a consequence of menopause, and alteration of the resident microbial community has been ascertained and investigated as a potential concurring cause of pathological conditions, such as vaginitis and urinary tract infections [16].

Notwithstanding the fact that follicular growth and oestrogen secretion occurs only twice yearly in dogs, the trophic effect of steroidal hormones on the lower uro-genital tract is well recognized in this species [1, 7].

Some complications are described as a consequence of sterilization. Urinary incontinence occurs due to lack of oestrogens and modification of the external urethral sphincter. In addition, modification of the vulva can lead to vulvar atrophy and cause perivulvar dermatitis [17]. In a study focused on verifying outcomes related to the timing of sterilization, the authors did not observe any difference in the incidence of the development of urinary incontinence for bitches sterilized in prepubertal age vs bitches undergoing sterilization at a later age in the 4 years after surgery [18].

In studies related to sterilization consequences, the effects of steroid hormone deprivation on vaginal mucosa conditions and bacteria colonization have not been investigated. This pilot study explores this rather complex field and applies methodologies, such as metataxonomic analysis, and previous comparable results are limited.

A limit of this investigation is the small number of animals and the possible inhomogeneity of the groups that included bitches of different breed, size and age. In addition, sterilized animals tended to be older than intact animals because elective surgery is not always performed at a very young age. Despite careful procedures to maintain sterility during sampling, we cannot exclude the possibility that bacterial contamination could have occurred in some conditions, especially in very small dogs or uncooperative animals.

Although cytological results suggested mucosal thinness and fragility in sterilized bitches, both metataxonomic investigation and culture techniques failed to discover strong differences in the microbial community colonizing the vaginal tract of either sterilized or intact bitches.

The lower number of different species isolated in sterilized bitches and the trend towards increased bacteria proliferation could be suggestive of an initial imbalance. However, in both groups of animals, the number of colony-forming units was consistently in the lower range, and this finding differs from that observed in clinical samples obtained from animals with vaginal exudates [13].

The metataxonomic approach highlighted a very high inter-individual variability, but no statistically significant difference between the different reproductive conditions were noted. Neither alpha nor beta measures were significantly different between the two groups; thus, no global characteristic could be identified that characterized these groups according to their condition.

At the phylum and class level, only Tenericutes and Mollicutes were significantly more abundant in anoestrus samples, and these microorganisms were only detected, by culture method, in one sample from the group of sterilized bitches.

Observing proportional abundance values, a discordance is noted between the mean and the median. This finding highlight that the identified prevalent genera were not evenly present in the samples in most cases. For example, *Photobacterium* had a considerable mean abundance among sterilized samples (14.03%), but this value was reduced when the median (3.42%) was used because this bacterium was found in very high percentages in only a few samples (e.g., S13 and S16) and in low quantity in all the other samples belonging to this group. On the other hand, *Salmonella*, *Mycoplasma* and *Staphylococcus* were present in moderate quantities in almost all the samples in both groups.

Many bacteria genera isolated in culture (*Staphylococcus, Streptococcus*, *Enterococcus*, *Proteus*) were also detected in the metataxonomic analysis; however, differences in relative abundance were noted. *Mycoplasma* was isolated from a single anoestrous bitch using culture techniques, whereas *Mycoplasma* resulted very widespread when genomic analysis was performed. Culture-independent methods allow for the identification of microorganism even in instances of minimal colonization or in cases that exhibited an inability or difficulty to grow in culture, so the results of the different techniques are not always comparable [19].

The genus *Mycoplasma* has special culture needs. Given the special organization and sample management procedures, the time that elapsed between the collection of samples and establishment of the culture could have compromised the viability of the *Mycoplasma* spp., thus preventing its detection in culture. In a previous work, the isolation of Mycoplasma from the vaginal tract did not vary between sterilized and intact bitches [20].

Quite surprisingly, the genus *Salmonella* was detected through sequencing in almost all the samples belonging to both groups of bitches. *Salmonella* is not part of the usual vaginal microbial flora of bitches [14, 20] and the genus was not even reported in previous investigations [15, 21]. In our study, bacteria belonging to the genus *Salmonella* did not grow in culture, the reason very likely being a very low microbial charge not sufficient to be detectable by culture methods, or a non-viable microorganism or even unsuitable culture conditions or last, the absence in culture of the ecological relationships that are typical of the microbial communities residing on biological samples [19].

Given that *Escherichia* and *Shigella* exhibit overlapping sequences [22], the metataxonomic analysis was unable to detect the genus *Escherichia*. Indeed, Greengenes resolves the issue of overlap leaving their sequences unclassified at levels below *Enterobacteriaceae* (https://greengenes.secondgenome.com/).

Culture-based techniques are believed to fail in detecting the majority of members of the resident microbial community: only viable bacteria that find appropriate growth conditions in culture can be isolated [15]. Our data from metataxonomic analysis do not confirm previous observations by Lyman et al. [15] who found two genera predominant in the bitch vagina, *Hydrotalea* and *Ralstonia.* It is difficult explain the presence of these two genera considering that the first group belongs to aquatic species, whereas the second is not commonly associated with animals [15]. In contrast to our investigation, the study was performed by amplifying the V4 region. This difference in techniques could explain the above-mentioned differences given that the choice of the hypervariable region has a well-known impact on metataxonomic results [23–27].

The relationship between the effect of time length of steroid hormone deprivation on the vaginal environment and the colonizing microorganisms deserves further investigations. In dogs, the absence of steroids stimulation is normal during anestrus, which lasts 3.5–4 months, so we expect that the effects of permanent hormonal deprivation would not be appreciable earlier than 3–4 months after sterilization. Gradual changes in the vaginal environment across the stages of menopausal transition are accompanied by modifications of the vaginal microbiota in women [6]. The vaginal bacteria communities of postmenopausal women, i.e. women without a menstrual cycle in the past 12 months [6, 16] show decreased proportions of lactobacilli*,* causing decreased lactic acid production and increased vaginal pH that possibly renders the vagina more susceptible to colonization by pathogenic bacteria and exacerbates the symptoms associated with vulvovaginal atrophy [16]. Indeed, lower relative abundance of *Lactobacillus* was also found in the bacterial community assemblage of postmenopausal women suffering from vulvovaginal atrophy, suggesting an alteration in vaginal microbiota homeostasis [6]. Bacteria of the genus *Lactobacillus* were detected in both groups of bitches by metataxonomic analysis regardless of their reproductive condition. Lactic acid-producing bacteria are the predominant colonizers of the vaginal tract of healthy women and are thought to be essential in maintaining urogenital health. A strong difference exists between the pH value of the vaginal tract of the bitch (approximately 7) and of women (approximately 4.5) [28]. This difference could explain why the microbiota composition is significantly different in the two species, and the role of lactic acid-producing bacteria is unclear in bitches [29].

## Conclusions

Further studies are necessary to confirm our results, obtained from a small number of animals. Although this investigation did not detect strong alterations in the vaginal bacteria community due to sterilization, it is worth noting that all the animals had been sterilized after puberty and that a relatively variable interval of time had elapsed since gonadal removal. Vaginal mucosa cytology was suggestive of dystrophic conditions. The clinical importance of our findings should be further assessed, and these studies should include bitches that have been sterilized for a longer and a less variable time.

## Methods

The work was performed on twenty-one healthy bitches of various breed, age and parity (Table 3) that had not undergone antimicrobial, probiotic or anti-inflammatory drug treatments over the last 6 months. The bitches were divided into two groups based on their reproductive condition: intact bitches in anoestrus (*N* = 11; mean age ± standard deviation (SD) 3.4 ± 2.2 years. Range: 1.5–7.5 years) and sterilized animals (*N* = 10; mean age ± SD 6.2 ± 3.4 years. Range: 1.5–12 years). A significant difference in age was noted between the two groups of animals (unpaired t test; *P* = 0.04). The reproductive history of the anoestrous bitches indicated that the last oestrus had occurred between 3 and 4 months before the experiments; the anoestrous phase was confirmed through serum progesterone concentration, which was measured using a chemiluminescent immunoassay (Immulite 2000®; Siemens Medical Solutions Diagnostics, Flanders, New Jersey, USA) and confirmed to be at a basal level (< 1 ng/ml). Time since sterilization ranged from 6 months to 4 years, and ovariohysterectomy was performed on all but three bitches. No bitch had been sterilized prior to 6 months of age.

**Table 3:** Age, breed and parity of anoestrous and sterilized bitches; type of surgery and years since surgery in the sterilized ones

	Breed	Age		Parity	Surgery type	Years from surgery
		Ys	Ms			
**Anoestrous bitches**						
1	Golden Retriever	2	1	0		
2	Lagotto	1	3	0		
3	Crossbreed	6	6	0		
4	Am.Staffordshire Terrier.	3	0	0		
5	Staffordshire Bull Terrier.	7	0	4		
6	English Bulldog	2	5	0		
7	Bloodhound	1	2	0		
8	Welsh Corgi	5	0	0		
9	Boxer	5	0	2		
10	Bolognese	1	5	0		
11	BelgianShepherd	2	5	0		
**Sterilized bitches**						
1	Newfoundland	8	1	1	OVH	2.5
2	Crossbreed	3	1	0	OV	2.5
3	Newfoundland	4	1	0	OVH	1
4	San Bernardo	1	6	0	OVH	1
5	Cocker Spaniel	3	3	0	OVH	2.5
6	Jack Russell Terrier	10	5	0	OVH	3
7	Cocker Spaniel	11	9	0	OVH	3
8	Crossbreed	5	0	0	OV	4
9	Cattle Dog	6	0	0	OVX	2
10	AustralianShepherd	9	0	3	OV	0.5

### Sample collection

All vaginal samples were collected from the middle vaginal vault after disinfection of the perivulvar area with povidone-iodine and through a sterile speculum. Two swabs were collected for metataxonomic analysis (Fecal Swab, Copan Italia, Brescia). A third swab was collected for bacteria culture (Transystem, Copan Italia, Brescia), and a fourth swab was collected for cytological examination.

The samples for metataxonomic analysis and culture were maintained at 4 °C for a maximum of 36 h before being processed [30, 31]. The swab for cytology was gently rolled over a microscope slide, air dried and stained with Diff Quick (Merck S.p.A., Milano, Italy).

### DNA extraction for microbiota analysis

Total DNA was extracted using a column-based kit (QIAamp DNA Mini Kit, QIAGEN) and 200 μl of vaginal content previously collected with the ‘Fecal Swab’ following the manufacturer’s instruction. In particular, thermal lysis was performed for 2 h, and RNaseA (100 mg/ml) was added to each sample to ensure an RNA-free preparation. Total DNA was resuspended in 200 μl of nuclease-free water and stored at − 20 °C until preparation for sequencing.

### 16S rDNA sequencing

The V3-V4 regions of *16S rRNA* gene were amplified with the primers CCTACGGGNGGCWGCAG (forward) and GACTACHVGGGTATCTAATCC (reverse) as described by Klindworth et al. [32]. After the initial amplification, the region was indexed, and samples were equimolarly pooled according to the 16S sequencing preparation guide (Illumina). The resulting library was sequenced on an Illumina MiSeq platform to obtain 300-bp long paired-end reads. A blank sample (i.e., with no DNA) was also included in the same sequencing run, which produced 50 quality-filtered reads.

### Microbiota and statistical analysis

Forward and reverse sequencing reads were pre-processed and assembled using the QIIME2 pipeline (version 2018.8) [33]. In particular, a de novo clustering procedure was performed for amplicon sequence variant (ASV) table construction using the DADA2 [34] bioinformatic tool plugin (parameters: --p-trunc-len-f 284, −-p-trunc-len-r 268). The taxonomic affiliation of each obtained ASV was determined using the Greengenes database (http://greengenes.lbl.gov, [35]) and a Naive Bayes classifier specifically trained on the target region selected for the present study.

Alpha indices (Richness and Pielou) and beta matrices (Bray-Curtis dissimilarity and weighted and unweighted UniFrac distances) were calculated for microbial community diversity analysis. The non-parametric Kruskal-Wallis test was used to compare alpha diversity between the two groups of bitches, whereas the Permanova test was used to compare the beta diversity parameters between the two groups. Additionally, beta diversity measures were used for ordination analysis with PCoA technique.

A differential abundance analysis was performed using ANCOM [36], a plugin available within QIIME2 framework, to assess differences in taxa abundances at each taxonomic level from ASVs to the phylum level.

A chi-square test was performed to assess differences in isolation rates of selected genera in anoestrus and sterilized bitches. *P* < 0.05 was considered statistically significant.

### Bacteria culture and statistical analysis

Bacterial isolation was performed according to standard lab culture techniques. Briefly, each swab was diluted in 1 ml of nutritive broth (Heart Infusion Broth, HIB, Conda, Madrid, Spain). Then, 10 μl and 100 μl from this suspension were used to inoculated different solid and liquid media, respectively, prior to incubation in different atmospheric conditions as follows: i) nutrient medium (Blood Agar Base n° 2, Biolife, Milan, Italy) with 5% defibrinated sheep blood (Allevamento Blood, Teramo, Italy) incubated at 37 °C for 24–48 h in aerobic, anaerobic and microaerophilic conditions (5–10%CO_2_); ii) the following selective media were also inoculated and incubated at 37 °C for 24–48 h under aerobic conditions: *Enterobacteriaceae* medium (McConkey agar, Oxoid, Basingstoke, UK), Bile-Esculin Azide Agar (BEA, Conda, Madrid, Spain), selective medium for *Clostridium perfringens* (TSC Agar Base, Biolife, Milan, Italy) and Fluid Thioglycollate medium (THG, Liofilchem, Roseto degli Abruzzi, TE Italy) at 37 °C for 24–48 h under anaerobic conditions.

Moreover, 200 μl of HIB broth suspension were filtered (0.45 μm), inoculated into *Mycoplasma* Experience® broth (1:10 dilution) and incubated for 7 days at 37 °C at 5% CO_2_. Then, 1 μl of *Mycoplasma* broth culture was plated in *Mycoplasma* Experience® medium and incubated for 7 days at 37 °C at 5% CO_2,_ with daily plate evaluation. All the microbial colonies grown on the first isolation agar media were counted and identified.

Genus identification of bacteria was phenotypically performed by macroscopic observation of colonies; Gram stain reaction; cellular morphology; growth on selective medium; catalase, oxidase, and mobility tests; and coagulase tube test. Species identification was performed by MALDI-TOF MS: Microflex LT instrument (MALDI Biotyper, Bruker Daltonics) equipped with FlexControl software (version 3.3, Bruker Daltonics).

Molecular analyses were performed to differentiate *Staphylococcus aureus* and *Staphylococcus pseudintermedius* [37, 38] when Maldi-TOF MS results were not discriminatory (score ≤ 2.3).

The colonies grown on the selective Mycoplasma medium (bacteria belonging to the *Mollicutes* group) were identified with 16S-rDNA-PCR-DGGE as described by McAuliffe et al. [39].

A chi-square test was performed to assess differences in isolation rates of selected genera in anoestrus and sterilized bitches. *P* < 0.05 was considered statistically significant.

## Supplementary Information

**Additional file 1.**

**Additional file 2.**

## Data Availability

The datasets used and/or analysed during the current study are available from the corresponding author on reasonable request.

## Abbreviations

AVS: Amplicon Sequence Variant
PCoA: Principal Coordinate Analysis
ANCOM: Analysis of Composition of Microbiomes
