# Supplementary figures and images for: Effect of sterilization on the canine vaginal microbiota: a pilot study
Source: BMC Vet Res. 2020 Nov 23;16:455. doi: 10.1186/s12917-020-02670-3 (PMC7684734; doi:10.1186/s12917-020-02670-3)

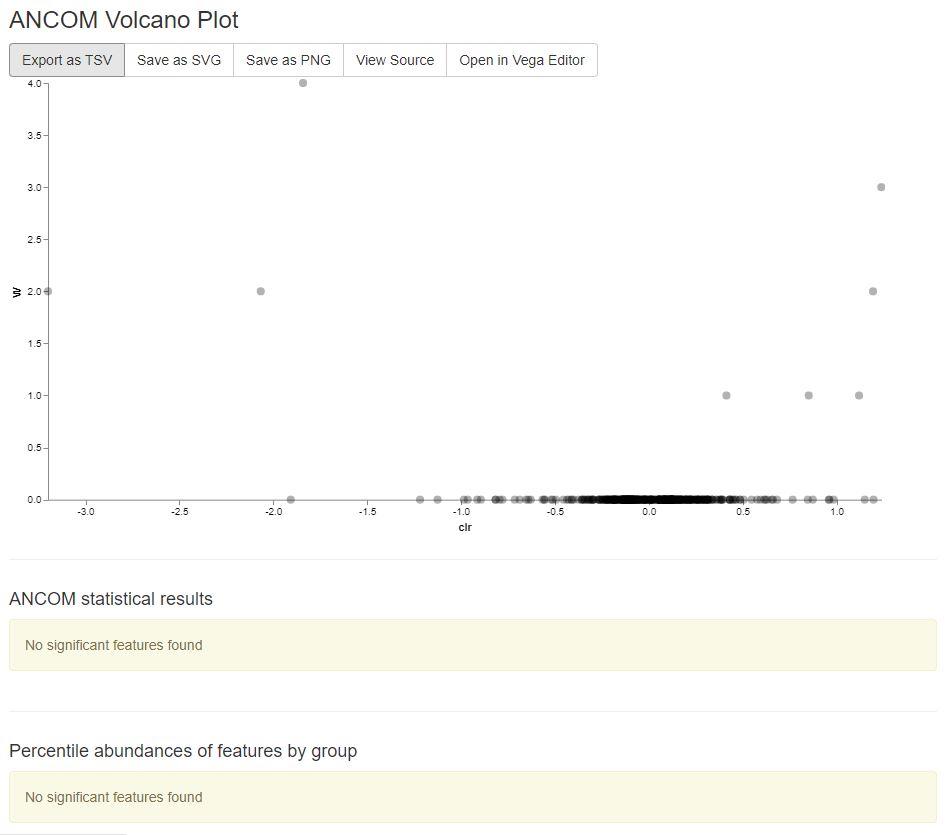

Supplement: Supplementary file 2 — Additional file 2. [file 12917_2020_2670_MOESM2_ESM.jpg]
